# Differences between international recommendations on breastfeeding in the presence of HIV and the attitudes and counselling messages of health workers in Lilongwe, Malawi

**DOI:** 10.1186/1746-4358-1-2

**Published:** 2006-03-09

**Authors:** Ellen G Piwoz, Yvonne Owens Ferguson, Margaret E Bentley, Amy L Corneli, Agnes Moses, Jacqueline Nkhoma, Beth Carlton Tohill, Beatrice Mtimuni, Yusuf Ahmed, Denise J Jamieson, Charles van der Horst, Peter Kazembe, the UNC Project BAN Study Team

**Affiliations:** 1 Academy for Educational Development, 1875 Connecticut Ave, NW, Washington, DC, 20009, USA; 2 University of North Carolina at Chapel Hill, 1700 Airport Road, CB# 3368 Chapel Hill, North Carolina, 27514, USA; 3 UNC Project, Private Bag A-106, Lilongwe, Malawi; 4 US Centers for Disease Control and Prevention, 1600 Clifton Road, NE, Atlanta, Georgia, 30333, USA; 5 Bunda College of Agriculture, Bunda, Malawi; 6 Kamuzu Central Hospital, Lilongwe, Malawi

## Abstract

**Background:**

To prevent postnatal transmission of HIV in settings where safe alternatives to breastfeeding are unavailable, the World Health Organization (WHO) recommends exclusive breastfeeding followed by early, rapid cessation of breastfeeding. Only limited data are available on the attitudes of health workers toward this recommendation and the impact of these attitudes on infant feeding counselling messages given to mothers.

**Methods:**

As part of the Breastfeeding, Antiretroviral, and Nutrition (BAN) clinical trial, we carried out an in-depth qualitative study of the attitudes, beliefs, and counselling messages of 19 health workers in Lilongwe, Malawi.

**Results:**

Although none of the workers had received formal training, several reported having counseled HIV-positive mothers about infant feeding. Health workers with counselling experience believed that HIV-infected mothers should breastfeed exclusively, rather than infant formula feed, citing poverty as the primary reason. Because of high levels of malnutrition, all the workers had concerns about early cessation of breastfeeding.

**Conclusion:**

Important differences were observed between the WHO recommendations and the attitudes and practices of the health workers. Understanding these differences is important for designing effective interventions.

## Background

In 2004, approximately 640,000 worldwide children became infected with HIV, primarily through mother-to-child (MTCT) transmission of the virus [1]. Transmission occurs in-utero, at the time of childbirth, and postnatally through breastfeeding. Programs to prevent MTCT provide HIV testing and counselling before birth, short-course antiretroviral prophylaxis for HIV-positive mothers, and modifications to standard obstetrical practices. Counselling about infant feeding is also included to prevent postnatal transmission of the virus.

International guidance currently states that when replacement feeding is acceptable, feasible, affordable, sustainable, and safe, avoidance of all breastfeeding by HIV-infected mothers is recommended to prevent postnatal transmission of HIV [2] (see Table 1). Otherwise, exclusive breastfeeding during the first months of life followed by early, rapid cessation of breastfeeding is recommended. The recommendations further state that HIV-infected mothers should receive counselling on the risks and benefits of different infant feeding options and be given guidance and support to choose the most appropriate option for their situation. In contrast, recommendations for infant feeding for HIV-negative women and women of unknown HIV status include exclusive breastfeeding for 6 months and continued breastfeeding for at least 2 years [3]. The Malawi Ministry of Health and Population recommends that HIV-positive mothers who breastfeed do so exclusively, but that breastfeeding be terminated early according to the mother's individual situation [4].

**Table 1:** WHO recommendations on HIV and Infant feeding

Topic	Recommendation
Infant feeding	When replacement feeding is acceptable, feasible, affordable, sustainable and safe, avoidance of all breastfeeding by HIV-infected mothers is recommended. Otherwise, exclusive breastfeeding is recommended during the first months of life.
	To minimize the risk of HIV transmission, breastfeeding should be discontinued as soon as feasible, taking into account local circumstances, the individual woman's situation and the risks of replacement feeding (including infections other than HIV and malnutrition).
	When HIV-infected mothers choose not to breastfeed from birth or stop breastfeeding later, they should be provided with specific guidance and support for at least the first 2 years of the child's life to ensure adequate replacement feeding. Programs should strive to improve conditions that will make replacement feeding safer for HIV-infected mothers and families.
Counselling on infant feeding	All HIV-infected mothers should receive counselling, which includes provision of general information about the risks and benefits of various infant feeding options, and specific guidance in selecting the option most likely to be suitable for their situation. Whatever a mother decides, she should be supported in her choice.
	Assessments should be conducted locally to identify the range of feeding options that are acceptable, feasible, affordable, sustainable and safe in a particular context.
	Information and education on mother-to-child transmission of HIV should be urgently directed to the general public, affected communities and families.

From "New data on the prevention of mother-to-child transmission of HIV and their policy implications: Conclusions and recommendations," by the WHO Technical Consultation on Behalf of the UNFPA/UNICEF/WHO/UNAIDS Inter-Agency Task Team on Mother-to-Child Transmission of HIV. Geneva: World Health Organization. Report No. WHO/RHR/01.28.

Exclusive breastfeeding is recommended for HIV-infected mothers who breastfeed because it protects against diarrhoea and other infections [5-7]. In addition, when compared with mixed feeding, exclusive breastfeeding was associated with reduced risk of HIV transmission in a study in South Africa [8] and with increased HIV-free survival in a study in Zimbabwe [9]. In the latter study, early mixed feeding was associated with a 4-fold increased risk of breastfeeding-associated HIV transmission at 6 months [9]. Several explanations for the increased risk of HIV transmission associated with early mixed feeding have been proposed, including increased gut inflammation and permeability to infection, higher viral load in breast milk, and more frequent breast health problems among mothers who mixed feed, but the causal mechanisms have not yet been identified [10].

Malawi is experiencing one of the worst HIV/AIDS epidemics in the world. Out of a total population of 12 million inhabitants, approximately 460,000 women and 83,000 children under age 15 are infected with HIV [11]. General health indicators in Malawi are also alarming. Nearly 50 percent of all children are chronically malnourished [12]. The country's infant mortality rate of 104 deaths per 1,000 live births is one of the highest in sub-Saharan Africa and is partially due to malnutrition and poor environmental conditions [12]. For HIV-infected mothers, high rates of malnutrition, contaminated water, and poor hygiene increase the risk of infant morbidity and mortality from replacement feeds, and thus safe options for infant feeding are limited.

Infant feeding practices in Malawi are far from optimal, and implementing recommendations for infant feeding among HIV-infected mothers in light of these patterns poses a challenge. For example, prolonged breastfeeding is common but a long duration of exclusive breastfeeding is not. The Malawi 2000 Demographic and Health Survey reported that more than half of all infants were continuing to breastfeed at 24 months, but the median duration of exclusive breastfeeding was just 2 months [12]. Thirty-one percent of 2–3 month-old infants and 80 percent of infants aged 4–5 months were already consuming solid foods [12].

In many sub-Saharan Africa countries, communities often take the recommendations of health providers as the final word [13,14]. In some countries, however, advice by health workers has contributed to sub-optimal feeding practices [15-17]. And yet, with formal training and supportive supervision, health workers can effectively increase rates of exclusive breastfeeding [17-22].

As key gatekeepers in influencing mothers' decisions on infant feeding [14,23], health workers can help to reduce rates of postnatal transmission of HIV and increase child survival by providing HIV-infected mothers with accurate information on infant feeding that captures the risks and benefits of different feeding options. Few studies, however, have reported on what health workers currently believe and practice regarding infant feeding for HIV-infected women. This is an important concern because attitudes and cultural beliefs may affect their counselling behaviour [24,25]. In this paper, we present data on Malawian health workers' attitudes and counselling messages relative to the current recommendations for infant feeding by HIV-infected mothers.

## Methods

Data were collected as part of a formative research study to inform the design of the Breastfeeding, Antiretroviral, and Nutrition (BAN) study, a clinical trial in Lilongwe, Malawi. The objectives of the BAN study are to investigate (a) the benefit of nutritional supplementation given to HIV-infected women during breastfeeding, (b) the benefit and safety of antiretroviral medications given either to infants or their HIV-infected mothers to prevent transmission during breastfeeding, and (c) the feasibility of exclusive breastfeeding followed by rapid cessation of breastfeeding at 6 months [26-28].

The formative study included in-depth individual interviews with HIV-infected mothers, health workers, community leaders and traditional birth attendants; observations of home environments with mothers of unknown HIV status; and focus group discussions with pregnant women, grandmothers, and fathers [29]. In this analysis we used data from in-depth individual interviews with 19 health workers who cared for pregnant women to assess the workers' attitudes about infant feeding in the presence of HIV, including their attitudes toward WHO and Malawian infant feeding recommendations, and the counselling messages they deliver to mothers. Health workers from area health centers in Lilongwe were recruited for the study. Field investigators conducted individual semi-structured interviews with health workers, asking a combination of set order questions and questions tied to health workers' responses. The interview guide, derived from extant literature and experiential data, included both closed (e.g., yes/no) and open-ended questions. Health workers were selected using purposive sampling techniques [30], with sampling from numerous geographical areas in Lilongwe in order to capture potential variations in attitudes and counselling messages concerning infant feeding.

All interviews were conducted in June and July 2002 by Malawian field investigators with prior training in nutrition or public health research. Before data collection, the field investigators participated in a 2-week training course on qualitative research methods and interviewing techniques [31]. They were also provided with the most recent clinical information on infant feeding and HIV/AIDS. The in-depth interview guides were developed in English, translated into Chichewa, the local language, and back-translated into English. All interviews were audiotaped in Chichewa and translated and transcribed verbatim for analysis.

Data were analyzed using methods described in Miles& Huberman [32]. First, data matrices were developed to analyze the health workers' responses to each interview question. Qualitative content analysis techniques [33] were used to identify major themes and sub-themes from the open-ended question responses. Throughout the analysis, we documented our analytic decisions using the audit trail methodology [34]. The frequency of responses to closed-ended questions is reported in absolute numbers and percentages. Direct quotes are used to describe the major themes and sub-themes from responses to the open-ended questions.

The institutional review boards at the U.S. Centers for Disease Control and Prevention, the University of North Carolina at Chapel Hill, and the National Health Science Research Committee in Malawi approved the formative research, and each participant provided written informed consent.

## Results

Slightly more than half of the 19 health workers were aged 30–39 years and 16 were women (Table 2). About three-quarters were in the nursing profession and almost all had trained 3–4 years in their occupation. First, we present findings on health workers' attitudes and counselling messages related to exclusive breastfeeding for mothers not known to be infected with HIV. Next, we specifically discuss the attitudes and counselling practices related to exclusive breastfeeding and early cessation of breastfeeding for HIV-infected mothers. A summary of the health workers' responses to closed-ended questions is presented in Table 3.

**Table 2:** Demographic characteristics of health workers (N = 19)

Characteristic	n
Gender	
Male	3
Female	16
Age (years)	
20–29	1
30–39	10
40–49	4
50–59	4
Occupation	
Nurse/Nurse Midwife	14
Clinical officer/medical assistant	5
Years of training	
1–2	1
3–4	17
5	1
Years in current occupation	
1–5	5
6–10	5
10–15	5
>16	4

**Table 3:** Health Workers' Attitudes and Counselling Messages Related to Infant Feeding (N = 19)

Characteristic	Question	Yes N (%)	No N (%)	Not Sure N (%)	No answer N (%)
General					
	Do you think that breast milk alone is enough food for a baby for the first 6 months of life?	18 (94)	1 (5)		
	Do you think it is possible for women in this community to breastfeed exclusively for 6 months?	11 (58)	7 (37)		2 (11)
	Do you think that it is possible for women to stop breastfeeding at 6 months in this community?	7 (37)	6 (32)	4 (21)	2 (11)
	Do you provide counselling on infant feeding?	17 (90)	2 (11)		
HIV specific					
	Do you believe that HIV can be transmitted from an HIV-infected mother to her child?	19 (100)			
	Do you believe that HIV can be transmitted through breastfeeding?	18 (95)	1 (5)		
	Have you ever counselled a woman about HIV and breastfeeding?	6 (32)	11 (58)		2 (11)
	Do you think a mother with HIV should breastfeed her baby?	10 (53)	7 (37)	1 (5)	1 (5)

### Attitudes and counselling messages related to exclusive breastfeeding for mothers not known to be infected with HIV

Almost all of the health workers (n = 18) knew and understood the correct definition of exclusive breastfeeding and reported believing that breast milk alone was sufficient for an infant during the first 6 months of life. One nurse midwife defined exclusive breastfeeding in this way, "It's the process whereby the child is only breastfeeding without giving him or her any food or water but breast milk only." Another nurse midwife explained why she thought breast milk was sufficient for the infant's first 6 months of life: "Because the child needs not much food and their body demands are still minimal. Breast milk contains all the food nutrients the baby needs."

Although the concept was understood, only 11 health workers believed that mothers in the community could practice exclusive breastfeeding for 6 months. Introduction of solid foods before 4 months was reported as the cultural norm and a major barrier to exclusive breastfeeding by seven health workers. One nurse's response illustrated this cultural phenomenon when she said, "They are used to introducing other foods to the baby very early, so they can't change." A nurse midwife also shared how culture hindered exclusive breastfeeding practices in her community: "Women do not understand. They say nowadays children are too hungry. Thus, many start supplementary food at less than 3 months. Some give infant formula to their child."

Health workers' attitudes are another barrier to exclusive breastfeeding. Although most had reported a belief that breast milk was sufficient for the infant's first 6 months of life, some did not appear to firmly believe this recommendation. For example, one nurse midwife illustrated how her professional practices conflicted with her personal beliefs when she said, "It [breast milk] is nutritious and contains all the nutrients. Ideally it is supposed to be adequate until the child is 6 months old...but sometimes it [breast milk] is not sufficient."

In terms of facilitators of exclusive breastfeeding, several health workers identified unemployment, as mothers who did not work had more time than employed mothers and were more likely to be with their infants. As noted by one nurse, "Most women are unemployed...so they have ample time to breastfeed." Another said, "Because you always have the child with you so milk is readily available."

Seventeen health workers reported direct experience counselling mothers on infant feeding, but the advice they provided was sometimes not consistent with WHO and national recommendations. One nurse midwife explained, "I tell mothers to practice exclusive breastfeeding up to 4 months without giving any supplementary feeds or water. From 4 months, I tell them to introduce supplementary feeds containing all the three food groups, so that when the baby is 6 months, it will not have problems digesting these foods since breast milk alone won't be enough." Another nurse advised mothers to "exclusively breastfeed for the whole year. Then I advise mothers to introduce nutritious foods to their babies."

### Attitudes and counselling messages related to feeding recommendations for HIV-infected mothers

Our analysis of the attitudes and counselling messages of the health workers relative to HIV-infected mothers focused on three major themes: (a) perceived risk to the infant of contracting HIV from breastfeeding, (b) differences in attitudes and counselling messages between health workers who had experience in HIV and infant feeding and those who did not, and (c) issues related to the early cessation of breastfeeding.

Almost all of the health workers interviewed (n = 18) were aware that breastfeeding was a mode of transmitting HIV from an infected mother to her infant (Table 3), but they were uncertain about the estimated risk from this practice. Eight believed that *all *or *most *HIV-infected mothers would transmit the virus during breastfeeding, while ten correctly believed that only *some *mothers would transmit the virus.

None of the health workers interviewed indicated having received any formal training in counselling HIV-infected mothers about feeding their infants. Nonetheless, six reported that they had experience in counselling such mothers on options for infant feeding. Most nurses with this experience believed that HIV-infected mothers should breastfeed exclusively, citing poverty as the primary reason why this was the most feasible option in their community. This sentiment was illustrated by a medical assistant who said, "Yes, HIV-infected mothers should breastfeed because they cannot buy infant formula." A nurse also shared this perspective: "Women with HIV should breastfeed because resources are scarce to buy infant formula in this community."

In addition to advising HIV-infected mothers to exclusively breastfeed, they also delivered messages of breast health to infected mothers who wanted to avoid transmitting the virus to their infants during breastfeeding. One nurse midwife counselled mothers to check their breasts and their infant's mouth for infections: "I advise them to make sure that the baby is kept away from [breast] infections and mouth sores." Similarly, another nurse midwife counselled HIV-infected mothers to use breast-milk substitutes when experiencing problems with breast health: "She should make sure that if she has breast sores, she should avoid breastfeeding her baby. Instead, bottle feed the baby if she can manage."

In contrast with the views of health workers with experience in counselling HIV-infected mothers, the 11 health workers reporting no direct experience were divided as to whether women with HIV should breastfeed at all. Seven believed that HIV-infected women should not breastfeed because of the risk involved in transmitting the virus to the infant. One nurse said: "Breast milk *always *contains HIV, so it will infect the baby." This perceived certainty of HIV transmission to an infant through breastfeeding most likely explained the statement of another nurse: "HIV-infected mothers should not breastfeed. They should use formula from the shops, even thin *phala *(porridge)."

The WHO recommendation for early, rapid cessation of breastfeeding was discussed. Health workers noted, however, that rather than counselling rapid cessation, they advised HIV-infected mothers to gradually introduce complementary foods after 6 months of exclusive breastfeeding. One nurse midwife said, "She should stop breastfeeding gradually. She should try giving the baby food and once it accepts it she can then stop the baby from latching." Agreeing, another nurse expressed her concern with programs recommending rapid or abrupt breastfeeding cessation: "With an HIV-positive mother it is difficult [to advise them] because it [breastfeeding cessation] is supposed to be done abruptly. But I think it should be done gradual while introducing supplementary feeds."

The recommendation for early cessation of breastfeeding for HIV-infected mothers conflicts with the cultural norm of breastfeeding for up to 2 years. Health workers were divided when asked whether it was possible for mothers in their community to stop breastfeeding at 6 months, as recommended in the BAN protocol, with seven responding "yes," six "no," four "possibly," and the remaining two with no answer. Explanations for early cessation of breastfeeding included pregnancy and maternal employment. One nurse midwife explained: "Some women become pregnant early [when the nursing baby is still young], so they feel it is not safe to continue breastfeeding the baby." As for stopping at 6 months because of employment status, a clinical officer stated: "Working mothers are more likely to stop breastfeeding their baby early."

Health workers identified concerns about the infant's nutritional status and food insecurity as barriers towards early cessation. This was illustrated by a nurse midwife who said, "The child is very young so it is difficult to stop breastfeeding because the baby develops malnutrition and has a lack of bonding with the mother. Food availability might also affect her from stopping breastfeeding because there is a shortage of food in this community."

The four health workers who indicated it was possible for women to stop breastfeeding at 6 months based their responses on the mother's HIV status and her concern for her infant's health. One nurse said, "If the [HIV-positive] mothers are told good reasons for stopping, they will listen and follow what they are advised." Similar to this response, a medical assistant mentioned that fear may influence HIV-infected mothers' infant-feeding behaviour when he stated, "HIV-positive mothers with a fear of transmitting HIV to their child could stop breastfeeding at 6 months."

## Discussion

Cultural norms affect health workers' attitudes and how they counsel mothers about complex behaviours. We undertook this study because existing data suggested that WHO and Malawian recommendations for infant feeding by HIV-positive mothers were in contradiction with local feeding norms and practices, and additional qualitative data were needed to better understand the challenges health workers face when counselling mothers about these recommendations. We observed that even without formal training, some experienced health workers were giving appropriate advice to HIV-infected mothers. However, misperceptions about breastfeeding and HIV were common, and attitudes and cultural beliefs influenced the counselling messages given on this subject, underscoring the importance of locally adapted messages and training on HIV and infant feeding.

Based on our findings, we developed a comprehensive training course for health workers in the BAN study, adapting existing training material [35-37] to local realities. The course focuses on developing core competencies in counselling for exclusive breastfeeding, early cessation, and replacement feeding after 6 months, and it addresses ways to respond to real and perceived constraints to safe feeding practices. In addition, we developed checklists and key messages to facilitate the counselling process. An evaluation of the counselling performance of health workers is underway.

Important changes to the BAN study protocol were also made [29], including a decision to counsel mothers to stop breastfeeding gradually between 6 and 7 months and to provide them with support before this time to prepare them for early cessation, as suggested by the health workers. This support includes counselling about ways to deal with family members' and communities' concerns for the infant who is not breastfed and the stigma that may come from not breastfeeding as well as introducing the infant to solid foods and cup-feeding before cessation. Because of high rates of malnutrition and food insecurity in the community, we also identified a locally produced and nutritious ready-to-use therapeutic food for non-breastfed infants after 6 months. Mothers are given a 6-month supply of the ready-to-use food and are counselled to feed 25 g (1 teaspoon) 3 times per day as a replacement for breast milk. This food is relatively low cost (US $2.50/kg or $6/month) and currently used for community-based management of severe malnutrition in Malawi [38]. Counselling on complementary feeding using family foods is also provided. The BAN study will test the feasibility and impact of replacement feeding using the ready-to-use food after 6 months in our study population of approximately 2400 HIV-positive mothers and their infants.

There are many factors influencing mothers' feeding decisions and, in this population, our larger formative study revealed that mothers place a high level of importance on the advice of health workers. Mothers trust the information they receive from these workers and believe it is accurate and beneficial for their infant's health. This creates a challenging situation for counselling on infant feeding because there are several aspects of HIV transmission through breastfeeding that are not yet well understood scientifically. This includes issues such as the mechanisms through which breastfeeding-related transmission occurs, how and when to stop breastfeeding to balance the competing risks of HIV transmission and mortality from other infectious diseases and malnutrition, and the safest way to feed infants who are no longer breastfeeding using locally available foods.

At present, in many countries health workers are expected to counsel HIV-positive mothers on infant feeding with little or no training, as reported in a recent evaluation in Malawi, Uganda, Kenya, and South Africa (draft report prepared for UNICEF by M. Chopra, [2005]: Report of the four country review of prevention of mother-to-child transmission of HIV [PMTCT] programmes). Thus, we encourage further research on how to counsel HIV-positive mothers about infant feeding, local adaptation of counselling messages, and rapid implementation of locally adapted training so that health workers have the skills needed to counsel mothers effectively about safe infant feeding. Failure to provide this training may compromise the health of infants if counselling is based on personal attitudes or otherwise subject to bias.

Regarding possible limitations of the present study, we note that this was a primarily a qualitative examination of the knowledge, attitudes, and counselling messages of Malawian health workers that was based on a small number of purposively selected respondents. Although informative for our purposes, the results cannot be generalized to the large population of nurses and other health workers working in Lilongwe. On the other hand, through our qualitative approach we were able to identify cultural norms on infant feeding, the attitudes of health workers toward existing recommendations, and deficits in the counselling messages of workers relative to infant feeding that may not have been captured through other methods.

## Conclusion

There are often important discrepancies between the attitudes of health workers, cultural beliefs and counselling messages, and existing recommendations on HIV and infant feeding. Qualitative research is useful for understanding these differences and for adapting counselling messages to the local context. Competency-based training on HIV and infant feeding is necessary in Malawi, and elsewhere, to strengthen health worker counselling and to ensure safe infant feeding practices. Additional data on the impact of this counselling on infant feeding practices and health outcomes are needed.

## Competing interests

The author(s) declare that they have no competing interest.

## Authors' contributions

EP contributed to designing the study protocol, data analysis and drafted the manuscript

YOF contributed to the data analysis and drafted the manuscript

MEB contributed to designing the study protocol and data analysis

ALC contributed to designing the study protocol, data analysis and supervised the data collection of the study

AM contributed to designing the study protocol and data collection

JN participated in data collection and critiqued drafts of the manuscript

BCT contributed to designing the study protocol and data analysis

BMM contributed to designing the study protocol and data collection

YA contributed to designing the study protocol and critiqued drafts of the manuscript

DJJ contributed to designing the study protocol and critiqued drafts of the manuscript

CvdH contributed to designing the study protocol, data analysis and critiqued drafts of the manuscript

PK contributed to designing the study protocol and critiqued drafts of the manuscript

All authors read and approved the final manuscript
